# Pericardial effusion is correlated with clinical outcome after pulmonary artery denervation for pulmonary arterial hypertension

**DOI:** 10.18632/oncotarget.14031

**Published:** 2016-12-20

**Authors:** Shao-Liang Chen, Hang Zhang, Du-Jiang Xie, Juan Zhang, Ling Zhou, Meng-Xuan Chen, Gregg W. Stone

**Affiliations:** ^1^ Division of Cardiology, Nanjing First Hospital, Nanjing Medical University, Nanjing, China; ^2^ Division of Cardiology, Nanjing Heart Center, Nanjing, China; ^3^ Emory College of Arts and Science, Atlanta, GA, USA; ^4^ Columbia University Medical Center and the Cardiovascular Research Foundation, New York, NY, USA

**Keywords:** pulmonary arterial hypertension, pericardial effusion, pulmonary artery denervation, clinical worse event, rehospitalization

## Abstract

**Objectives:**

Pericardial effusion (PE) is correlated with outcomes in patients with pulmonary arterial hypertension (PAH). Pulmonary artery denervation (PADN) was used for treatment of PAH. The present study aimed to analyze the prognostic value of PE for outcomes after PADN in patients with WHO Group I, Group II and Group IV PAH.

**Results:**

PE, frequently seen in patients with connective tissue disease, was featured by fast heart rate, decreased exercise capacity, more syncope, worsening pulmonary arterial hemodynamic and right atrium size. PADN procedure resulted in dramatic reduction of PE. After a median of 376 days follow-up, the rate of PAH-related event, all-cause death and rehospitalization increased over the PE amount and occurred in 29.8%, 19.7% and 25.2% of patients with PE, different to 3.4%, 3.4% and 6.8% of patients without PE (*p* = 0.034, *p* = 0.041 and *p* = 0.039, respectively). The reduction of PE during follow-up was similar among three groups.

**Materials and methods:**

Between March 2012 and July 2014, a total of 66 consecutive patients (52 ± 16 years) who underwent PADN were stratified by no PE (*n* = 20), PE < 10 mm (*n* = 29) and PE ≥ 10 mm (*n* = 17) according to baseline echocardiograph. Dynamic change of PE and its correlation with PAH-related event after PADN were measured.

**Conclusions:**

PE is associated with increased PAH-related event after PADN. PADN results in significant similar reduction of PE among patients with Group I, Group II and Group IV PAH.

## INTRODUCTION

Pulmonary artery hypertension (PAH) is a debilitating condition, resulting in dyspnea and fatigue, impaired exercise capacity, and reduced survival [1]. When PAH develops, right ventricular (RV) and right atrial pressure (RAP) increased progressively, the fluid reabsorption of pericardial fluid via the venous or lymphatic channels draining into the right atrium is damaged, [2–6] leading to pericardial effusion (PE). The prevalence of PE varies from 21% to 29%, and higher in patients with connective tissue disease (CTD) [4, 6]. Pericardial fluid accumulation masks the echocardiographic findings of cardiac tamponade [6] and impairs the ventricular diastolic filling, which carries an additional risk to patients with PAH, [4, 6] particularly in PAH due to CTD [7]. As a result, PE in PAH is an indicator of right heart failure and poor prognosis.

Our group recently, reported the actual experimental [8] and short-term clinical results of pulmonary artery denervation (PADN) via significant reductions in pulmonary arterial pressure (PAP) and PE size, and improvement of 6MWD at 3-month follow-up in patients with idiopathic PAH who were unresponsive to medication [9]. We also found that the presence of PE was associated with less reduction of PAP [9]. However, the correlation of PE with outcomes after PADN remained to be unclear. Accordingly, the present study aimed to analyze the dynamic change of PE and its predictive value for clinical outcomes in consecutive patients with idiopathic or secondary PAH or secondary PH from left ventricular dysfunction [LVD] who underwent PADN procedure.

## RESULTS

### Patient population

A total of 70 patients with qualifying PAH/PH were screened for this study. Four patients were excluded: 2 patients could not lie flat for 20 minutes, 1 died on the ward after consent while waiting for the procedure, and 1 died from temporary pacing catheter-induced RV perforation before the PADN procedure. The study population thus consisted of 66 patients for whom a PADN procedure was attempted: 21 idiopathic PAHs; 19 PHs due to LVD (16 due to prior myocardial infarction and 3 dilated cardiomyopathy); 10 CTDs (6 with systemic lupus erythematosus, 2 with mixed connective tissue disease and 2 with primary Sjogren's syndrome); 9 CTEs; and CHDSR in 8 patients (5 patients with atrial septal defect and 3 patients with patent ductus arteriosus which were repaired surgically). Finally, there were 39 patients in WHO Group I, 19 in Group II and 9 in Group IV according to WHO classification.

### Baseline clinical characteristics

Compared to patients without PE, those with PE had more CTD (24.3% vs. 3.4%, *p* = 0.038, Table 1). Increased heart rate and WHO functional class 3–4, presentation of syncope, and decrease of 6MWD demonstrated a stepwise fashion across increasing baseline PE amount. Particularly, more prostacyclin analogues were used in overall patients (68.2%) based on non-PAH expert.

**Table 1:** Baseline clinical variables in all patients

	Overall(*n* = 66)	No effusion(*n* = 29)	Effusion< 1 cm(*n* = 20)	Effusion≥ 1 cm(*n* = 17)	*P* valuetrends
**Age**, yr	51.7 ± 15.7	50.3 ± 16.9	56.7 ± 12.3	48.3 ± 16.7	0.674
**Female**, n (%)	39(59.1)	19(65.5)	12(60.0)	8(47.1)	0.451
**Height**, cm	163.8 ± 7.9	162.9 ± 7.9	162.8 ± 8.5	166.6 ± 7.1	0.240
**Weight**, kg	60.6 ± 10.9	60.9 ± 10.4	58.6 ± 10.3	62.5 ± 12.8	0.552
**Blood pressure**, mmHg					
Systolic	125.6 ± 15.7	125.4 ± 13.6	126.7 ± 18.2	124.8 ± 16.7	0.933
Diastolic	81.3 ± 12.1	79.9 ± 8.6	80.5 ± 12.9	84.4 ± 15.9	0.463
**Heart rate**, bpm	77.7 ± 13.7	73.5 ± 12.5	78.5 ± 12.8	83.9 ± 15.0	0.041
**NT-pro BNP**, pg/ml	2206 ± 2257	1949 ± 3194	1751 ± 1838	3237 ± 3219	0.237
**Borg Index**, scores	2.43 ± 1.23	2.43 ± 1.32	2.35 ± 1.26	2.53 ± 1.11	0.910
Class stratification, n (%)					0.531
≤ Class 3	53(80.3)	24(82.8)	15(75.0)	14(82.4)	
Class 4–5	13(19.7)	5(17.2)	5(25.0)	3(17.6)	
**WHO functional class**, points	2.68 ± 0.64	2.52 ± 0.63	2.75 ± 0.44	2.88 ± 0.78	0.146
Class stratification, n (%)					0.012
Class 1–2	23(34.8)	12(41.4)	5(25.0)	6(35.3)	
Class 3–4	43(65.2)	17(58.6)	15(75.0)	11(63.7)	
**6-minute walk distance**, m	357.6 ± 117.9	389.3 ± 119.8	354.3 ± 118.3	307.2 ± 101.6	0.022
**Etiologies**, n (%)					
Idiopathic PAH	21(31.8)	9(31.0)	3(15.0)	9(52.9)	0.076
Connective tissue disease	10(15.2)	1(3.4)	5(25.0)	4(23.5)	0.038
CHD surgical repair	8(12.1)	5(17.2)	2(10.0)	1(5.9)	0.492
Left heart dysfunction	19(28.8)	10(34.5)	6(30.0)	3(17.6)	0.472
CTEPH	9(13.6)	3(10.3)	5(25.0)	1(5.9)	0.189
**Time to diagnosis of PAH**, yr	3.80 ± 0.65	4.31 ± 1.14	3.42 ± 1.28	3.38 ± 0.67	0.791
**Presentation at enrollment**, n (%)					
Syncope	14(21.2)	1(3.4)	4(20.0)	9(52.9)	< 0.001
Fatigue	62(93.9)	27(93.1)	18(90.0)	16(94.1)	0.880
Chest pain	14(21.2)	6(20.7)	4(23.5)	4(23.5)	0.892
Dyspnea	65(98.5)	28(96.6)	20(100.0)	17(100.0)	0.877
**Medication before PADN**, n (%)					
Calcium channel antagonist	7(10.6)	4(13.8)	2(10.0)	1(5.9)	0.698
ERA	14(21.2)	6(20.7)	4(20.0)	4(23.5)	0.860
Prostacyclin	45(68.2)	20(69.0)	11(55.0)	14(82.4)	0.274
5′-PDE	4(6.1)	1(3.4)	1(5.0)	2(11.8)	0.337
Diuretics	48(72.7)	19(65.5)	15(75.0)	14(82.4)	0.174
Digoxin	22(33.3)	10(34.5)	6(30.0)	6(35.3)	0.925

NT, N-terminal; BNP, brain natriuretic peptide; PAH, pulmonary arterial hypertension; CHD, congenital heart disease; CTEPH, chronic thromboembolitic pulmonary hypertension; ERA, endothelin receptor antagonist; 5′-PDE, phosphodiesterase type-5 inhibitors

### Measurements by echocardiograph and right heart catheterization

Moderate to severe PE was associated with higher PAP, RAP, RVP, PVR, Tei index, and lower CO (Table 2), compared to no or mild PE. Notably, RA area increased in a stepwise fashion across increasing the amount of PE (*p* < 0.001, Table 2).

**Table 2:** Measurements by cardiac echo and right heart catheterization in all patients

	Overall(*n* = 66)	No effusion(*n* = 29)	Effusion< 1 cm(*n* = 20)	Effusion≥ 1 cm(*n* = 17)	*P* valuetrends
**Echocardiographic measurements**					
LADs, mm	41.9 ± 8.9	40.3 ± 7.6	44.9 ± 9.2	40.9 ± 10.2	0.188
LVDd, mm	44.6 ± 12.2	46.1 ± 12.4	44.6 ± 10.1	42.0 ± 14.2	0.543
LVDs, mm	29.9 ± 12.5	31.4 ± 13.3	29.7 ± 9.1	27.5 ± 14.7	0.606
LVEF, %	62.5 ± 12.3	61.5 ± 14.6	62.2 ± 8.2	64.8 ± 12.5	0.672
Systolic PA pressure, mmHg	92.8 ± 30.8	85.5 ± 34.8	88.7 ± 22.3	110.0 ± 26.7	0.023
Mean PA pressure, mmHg	42.3 ± 15.9	45.3 ± 16.4	39.1 ± 12.9	40.8 ± 13.2	0.531
Systolic RV pressure, mmHg	91.8 ± 30.5	83.9 ± 33.4	87.7 ± 22.8	110.0 ± 26.6	0.013
Mean RA pressure, mmHg	12.0 ± 6.4	10.3 ± 3.5	11.9 ± 3.6	15.0 ± 5.4	0.018
RA diameter, mm					
Long-axis	58.6 ± 11.2	53.2 ± 9.7	61.1 ± 11.6	65.1 ± 9.2	0.001
Short-axis	49.3 ± 10.6	44.1 ± 7.9	51.6 ± 9.8	55.6 ± 11.6	0.001
RA area, cm^2^	23.4 ± 9.1	18.8 ± 6.0	25.4 ± 9.2	29.0 ± 9.5	< 0.001
Pericardial fluid, mm	2.75 ± 3.39	0	5.49 ± 1.29	11.76 ± 1.08	< 0.001
Tei	0.63 ± 0.15	0.59 ± 0.16	0.62 ± 0.15	0.71 ± 0.14	0.039
**Measurements by RHC**					
Systolic PA pressure, mmHg	87.9 ± 29.8	85.4 ± 35.4	88.5 ± 17.9	103.5 ± 25.2	0.029
Mean PA pressure, mmHg	53.1 ± 19.1	51.7 ± 22.4	57.8 ± 11.6	61.8 ± 17.7	0.039
PCWP, mmHg	14.8 ± 11.5	15.6 ± 12.7	13.1 ± 9.0	15.4 ± 12.5	0.736
Mean RA pressure, mmHg	12.6 ± 7.2	11.5 ± 7.9	11.8 ± 6.2	16.5 ± 5.8	0.031
Systolic RV pressure, mmHg	86.3 ± 29.0	82.8 ± 35.3	84.5 ± 17.6	97.8 ± 26.1	0.033
PVR, Wood Units	13.8 ± 8.6	12.8 ± 6.8	13.3 ± 7.5	18.6 ± 12.4	0.036
Cardiac output, L/min	3.26 ± 1.25	3.26 ± 1.32	3.21 ± 1.15	2.66 ± 1.23	0.042

LADs, left atrial diameter at end-systole; LVDd, left ventricular diameter at end-diastole; LVDs, left ventricular diameter at end-systole; LVEF, left ventricular eject fraction; PA, pulmonary artery; RV, right ventricular; RA, right atrial; PCWP, pulmonary capillary wedge pressure; PVR, pulmonary vessel resistance.

**Figure 1:**
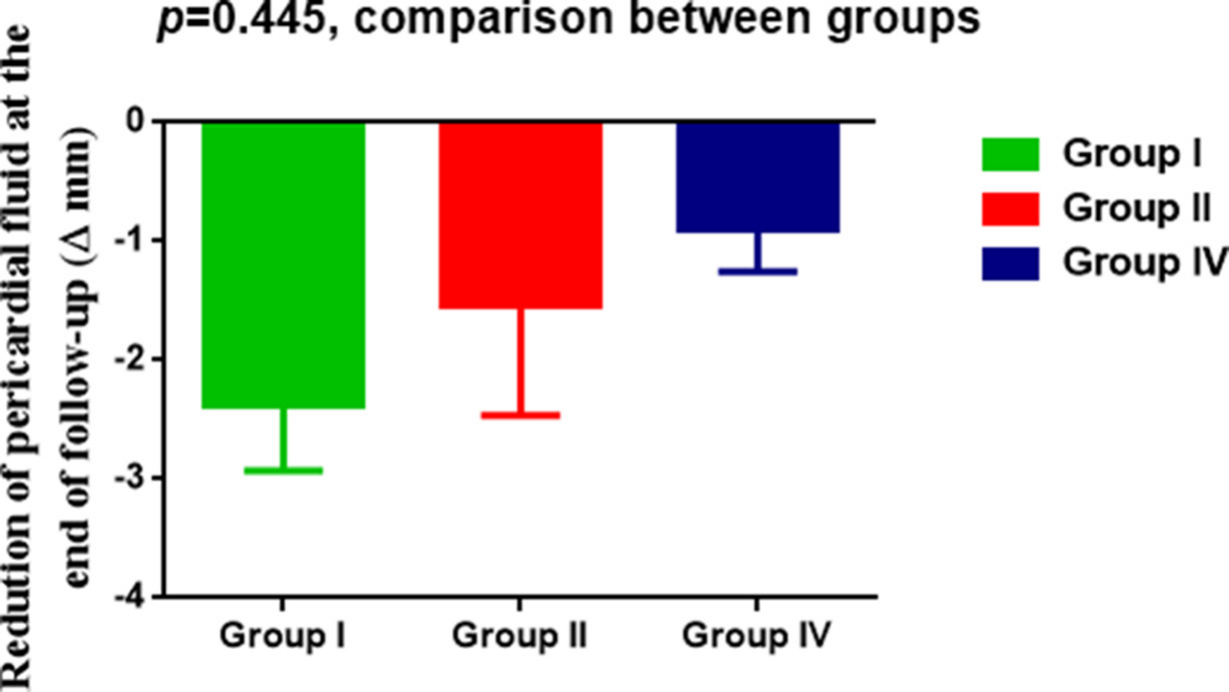
Dynamic change of pericardial effusion (PE) PADN was associated with significant reduction of PE in three groups classified by WHO definition.

As shown in Table 3, a greater mean absolute reduction of mPAP and sPAP just post-PADN was achieved in patients without PE (−12.1 mmHg and −12.3 mmHg ), when compared to patients with PE ≥ 1 cm (−4.0 mmHg and −6.3 mmHg) and patients with PE < 1 cm (−4.3 mmHg and −8.8 mmHg, *p* = 0.017 and *p* = 0.023, respectively). After 24 h post-PADN, the reduction of sPAP and mPAP was comparable between three groups, a finding in consistent with the dynamic change of pericardial fluid amount (Figure 1): less reduction just post-PADN, almost 50% reduction at 24 h after PADN treatment (*p* < 0.001). Since then, gradual absorption of PE over the time was associated with greater reduction of sPAP and mPAP in patients with PE (Table 3). The reduction of PE in the Group I seemed to be great but non-significant to that in Group II and Group IV (Figure 1), leading to similar improvement of hemodynamic between groups (data not showed here).

**Table 3:** Dynamic change of hemodynamic parameters measured by right catheterization

	Overall (*n* = 66)	No effusion (*n* = 29)	Effusion < 1 cm (*n* = 20)	Effusion ≥ 1 cm (*n* = 17)	*P* value trends
**Reduction of systolic PAP**					
**Post-PADN**					
Absolute value, mmHg	−8 ± 10	−12.3 ± 13.6	−8.8 ± 11.4	−6.3 ± 8.0	0.023
Percentage, %	−11 ± 12	−14.8 ± 13.5	−10.8 ± 10.2	−6.2 ± 7.5	0.036
**24 h**					
Absolute value, mmHg	−9.6 ± 13	−12.4 ± 18.2	−10.2 ± 12.7	−8.7 ± 11.4	0.070
Percentage, %	−11 ± 16	−13.5 ± 15.8	−11.4 ± 18.7	−10.9 ± 13.5	0.993
**End of follow-up**					
Absolute value, mmHg	−17.9 ± 14.5	−13.5 ± 9.1	−18.8 ± 16.1	−21.3 ± 16.2	0.242
Percentage, %	−20.4 ± 13.9	−14.6 ± 16.3	−20.1 ± 21.2*	−22.8 ± 23.1*	0.563
**Reduction of mean PAP**					
**Post-PADN**					
Absolute value, mmHg	−5 ± 11	−12.1 ± 12.5	−4.3 ± 5.7	−4.0 ± 5.1	0.017
Percentage, %	−12 ± 19	−22.7 ± 20.2	−7.7 ± 12.0	−6.5 ± 18.8	0.006
**24 h**					
Absolute value, mmHg	−6.6 ± 10	−9.3 ± 13.1	−6.8 ± 7.9	−4.8 ± 6.4	0.052
Percentage, %	−13 ± 18	−19.5 ± 21.6	−12.5 ± 12.4	−7.7 ± 17.3	0.048
**End of follow-up**					
Absolute value, mmHg	−10.5 ± 8.5	−9.4 ± 8.8	−11.6 ± 8.7	−11.1 ± 8.1	0.657
Percentage, %	−19.3 ± 20.6	−19.7 ± 14.4	−20.3 ± 21.6*	−19.1 ± 20.4*	0.218

*p < 0.01, compared to the percentage of reduction post-PADN.

PAP, pulmonary arterial pressure; PADN, pulmonary artery denervation.

Of 37 patients with PE, complete disappearance of PE at the end of follow-up was seen in 4 patients; PE increased to near baseline level in 3 patients who had significantly reduced PE at 3 month after PADN, these 3 patients died thereafter. Of 29 patients without PE, newly appearance of PE was seen in 1 patient.

### Morbidity and mortality

**Figure 2:**
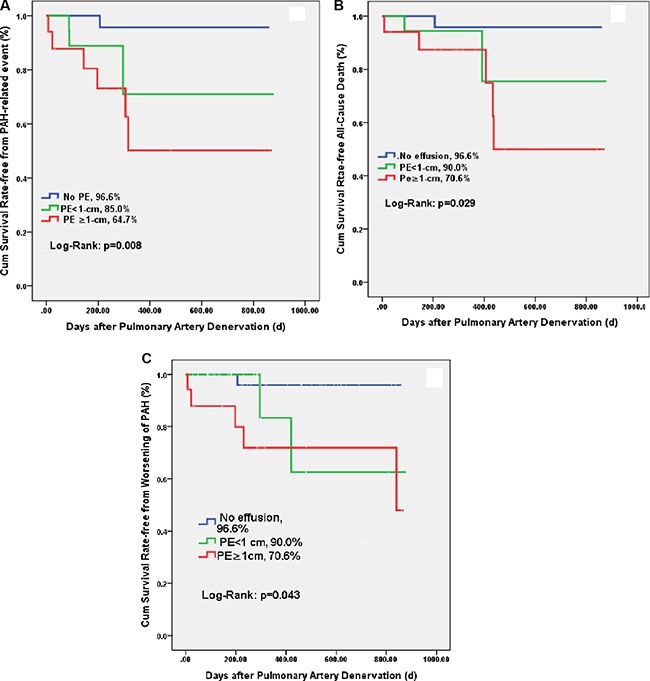
Kaplan-Meier survival analysis Patients with moderate and severe pericardial effusion had lower PAH-related event-free survival rate (**A**) and all-cause-free survival rate (**B**) and worsening of PAH (**C**).

Median (interquartile range) follow-up was 376(307–999) days. As shown in Table 4 and Figure 2, PAH-related adverse events occurred in 10 (15.2%) patients during follow-up, including 1 patient without PE, 3 mild PE and 6 moderate/severe PE (3.3% vs. 15.0% vs. 35.3%, *p* = 0.008, Figure 2A). Most events occurred within one-year. There was no procedure-related complication.

**Table 4:** Clinical outcomes during follow-up

	Overall (*n* = 66)	No effusion (*n* = 29)	Effusion < 1 cm (*n* = 20)	Effusion ≥ 1 cm (*n* = 17)	*P* value trends
**Follow-up** (d, median [IQR])	376 (307−999)	374 (309−980)	380 (307−999)	377 (310−911)	0.908
**PAH-related events**, *n* (%)	10 (15.2)	1 (3.4)	3 (15.0)	6 (35.3)	0.015
Death	6 (9.1)	0	2 (10.0)	4 (23.3)	0.044
Atrial septostomy	0	0	0	0	NS
In list for lung transplant	2 (3.0)	0	0	2 (11.8)	0.051
Needing IV or SQ drugs	6 (9.1)	0	1 (5.0)	5 (29.4)	0.003
Worsening of PAH	8 (12.1)	1 (3.4)	2 (10.0)	5 (29.4)	0.032
6MWD decline by > 15%	8 (12.1)	1 (3.4))	2 (10.0)	5 (29.4)	0.032
Worsening of symptoms^#^	8 (12.1)	1 (3.4)	2 (10.0)	5 (29.4)	0.032
Additional treatments	14 (21.2)	3 (10.3)	4 (20.0)	7 (41.2)	0.047
**All-cause death**, *n*(%)	8 (12.1)	1 (3.4)	2 (10.0)	5 (29.4)	0.032
**Rehospitalization**, *n* (%)	11 (16.7)	2 (6.8)	3 (15.0)	6 (35.3)	0.037

IQR, interquartile range; PAH, pulmonary arterial hypertension; IV, intravenous; SQ, subcutaneous; 6MWD, 6-minute walk distance.

^#^defined as a change from baseline to a higher WHO functional class (or no change in patients who were in WHO functional class IV at baseline) plus the appearance or worsening signs of right heart failure not response to oral diuretics therapy.

All-cause mortality occurred in 8 patients (12.1%) during follow-up, with a significant different rate in those without PE (3.4%), with mild PE (10%) and with moderate to severe PE (29.4%, *p* = 0.032, Figure 2B). The rate of PAH-related event was comparable between patients with Group I, Group II and Group IV PAH (Data not showed here). Similarly, patients with PE had increased rate of the worsening of PAH (Figure 2C).

### Univariate analysis

As shown in Table 5, univariate analysis showed that patients who had PAH-related event or all-cause death had increased PAP and systolic RVP, and RAP estimated by echo, more pericardial fluid, and decreased 6MWD.

**Table 5:** Univariate analysis of the associations with PAH-related event and all-cause death

	For PAH-related event		For All-cause death	
	95% CI of difference	*P* value	95% CI of difference	*P* value
**Variables by RHC**				
Systolic PAP	−40.992∽−1.191	0.038	−46.248∽−2.701	0.021
Mean PAP	−29.241∽−4.261	0.009	−32.629∽−5.281	0.007
Mean RAP	−9.590∽−0.098	0.055	−9.967∽0.746	0.090
Systolic RV pressure	−40.084∽−1.348	0.036	−44.218∽−1.689	0.035
**Variables by echo**				
Systolic RV pressure	−46.079∽−6.042	0.012	−52.111∽−8.398	0.007
Systolic PAP	−43.748∽−2.787	0.027	−49.542∽−4.777	0.018
Mean RA pressure	−8.912∽−0.445	0.031	−10.137∽−0.889	0.020
Pericardial effusion	−7.198∽−1.456	0.004	−7.453∽−1.057	0.001
**6-minute walk distance**	16.154∽172.324	0.019	3.688∽177.097	0.041

HRC, right heart catheterization; PAP, pulmonary arterial hypertension; RAP, right atrial pressure; RV, right ventricular

## DISCUSSION

The current study for the first time reports the correlation of baseline pericardial effusion with clinical outcome after PADN in patients with different etiologies of PAH. The major findings are: (1) the presence of PE is the mark of the severity of PAH; (2) PADN led to significant reduction of PE, with subsequent less PAH-related event, all-cause death and worsening of PAH.

### Mechanism of PE in PAH

The echocardiographic factors that adversely affect prognosis in PAH are RV dysfunction and the presence of PE [2, 3]. When PAH develops, a higher RAP that limits the drainage of pericardial veins into the RA. On the other hand, inflammatory conditions, such as systemic lupus erythematosus and scleroderma, can independently affect the pericardium and lead to PE, [4, 6] in line with our finding that more patients with PE had CTD. Previous studies [2–6] have clearly showed that the presence or persistence of PE in PAH despite vasoactive therapy predicted worse outcomes, a result further supported by our finding that patients with PE had increased worse clinical events after PADN. Given the prognostic value of PE, the absence or resolution of PE with novel therapy should have suggested better prognosis [6].

### Sympathetic nerves, PE and PADN

The causality of PE and sympathetic nervous activity in the setting of PAH remains to be unknown. In general, the over activation of sympathetic nerves was evidenced by increased circulating catecholamine levels, [10, 11] abnormally high muscle sympathetic nerve activity (MSNA) [12, 13] and impaired heart rate variability [14]. PA is innervated by complex network of sympathetic nerves [15, 16] but its distribution in human body is under studied. Our unpublished experimental study showed that main trunk of sympathetic nerves was close to and paralleled to main stem of PA, an anatomic feature favors the performance of percutaneous intraluminal intervention [9].

Our finding that > 10% reduction of PAP on-treatment could be explained by the inhibitory effect of PADN on vessel constriction induced by overactivated sympathetic nerves, as PADN-induced sympathetic nerves injury could abolish the increase of PAP in an acute PH model resulted from balloon-distention.8 Furthermore, the reduction of PAP in a stepwise fashion across the crossing the baseline amount of PE implied the complicated interplay between PE and PAP. Taking together, overactivated sympathetic nerves activity played a critical role in maintaining the vicious circle involving RV failure, increased PAP and RAP, and accumulation of pericardial fluid.

Risk models for patients with PAH treated by medications have been established [7, 17]. The present univariate analysis showed that PAP, estimated RAP by echo, PE, and 6MWD correlated with PAH-related event and mortality after PADN, in line with previous studies using medication [18–21]. This could be at least partially explained by the greater reduction of PAP, with resultant resolution of PE, achieved by PADN procedure.

### Limitations

Small patient size was the first limitation. However, our results provided stronger evidences showing the prognostic value of PE for outcomes after PADN. Next, our results came from a single-center, and bias could be excluded. As an emerging treatment for PAH, even though PADN was only performed in several global tertiary centers, our data was recorded from real patients underwent PADN procedures. Finally, we did not analyze the correlation of PE with already established risk model. However, our data clearly showed that size of PE was the only predictor of clinical outcome after PADN.

## MATERIALS AND METHODS

### Patient population

Between March 2012 and July 2014, patients with either idiopathic PAH or PH arising from chronic thromboemboli (CTE) after surgery, CTD, from congenital heart disease after surgical repair (CHDSR), or secondary from left ventricular dysfunction (LVD) were considered for possible treatment with PADN, if their resting mean PAP (mPAP) ≥ 25 mmHg (for all patients) and pulmonary vascular resistance (PVR) ≥ 3 Wood Units (for PH secondary from LVD) measured by right heart catheterization (RHC). Exclusion criteria included active infection, cancer, toxin- or anorexia-induced PH, portal hypertension, CTEPH without surgical treatment, intolerable to PADN procedure and inability to provide consent. The study was approved by the Institutional Review Board and Ethics Committee of the Nanjing First Hospital (Nanjing, China), and all patients provided written inform consent.

### RHC measurements

Resting RAP, RV pressure, PAP, pulmonary artery occlusion pressure (PAOP) or left ventricular end-diastolic pressure, [1, 22, 23] and cardiac output (CO) were obtained with a 7F flow-directed Swan-Ganz catheter. PVR ([mean PAP-PAOP]/CO) was then derived. All measurements were taken at end-expiration.

### PADN procedure

PADN was performed using a dedicated 7-F temperature-sensing ablation catheter. The details of the device and PADN procedure have been previously described [8, 9]. Briefly, PADN was performed only in the peri-conjunctional area between the distal main trunk and ostial left branch.

### Peri-procedural medication

Following the procedure, oral warfarin was prescribed and adjusted to an International Normalized Ratio (INR) of 1.5–2.5 for all patients. Aspirin (100 mg/d) and clopidogrel (75 mg/d) were indefinitely prescribed in the presence of contraindications for warfarin.

### Assessment of functional capacity

Functional capacity was determined using a standard 6-minute walk distance (6MWD), [1] Borg scale, [24] and the World Health Organization (WHO) functional classification [1] by a physician blinded to the study design.

### Echocardiographic measurements

Transthoracic echocardiography was performed and analyzed at the Nanjing Medical University Echocardiographic Laboratory. Measurements were performed according to American Society of Echocardiography guidelines. [25, 26] Digital echocardiographic data that contained a minimum of 3 consecutive beats (or 5 beats in cases of atrial fibrillation) were acquired and stored. Systolic RVP was set equal to systolic PAP (sPAP) in the absence of pulmonary stenosis. sPAP was calculated as the sum of RAP and the RV to RA pressure gradient during systole. RAP was estimated based on the echocardiographic features of the inferior vena cava and assigned a standard value. The RV to RA pressure gradient was calculated as 4 vt^2^ using the modified Bernoulli equation. The mPAP was estimated according to the velocity of the pulmonary regurgitation jet in m/s. The tricuspid excursion index (Tei) was defined as (A–B)/B, where A was the time interval between the end and the onset of tricuspid annular diastolic velocity, and B was the duration of tricuspid annular systolic velocity (or the RV ejection time) [26]. RA area was defined as πab, where a was the long semi-radius of RA, and b was the short semi-radius. [25, 26] Pericardial effusion severity was characterized as trace to small if the pericardial space separated by < 1 cm in diastole in any plane. Moderate or greater effusion was defined as pericardial space separation of ≥ 1 cm during diastole described previously [2].

### Definitions

PAH-related clinical events, defined as those caused by worsening of PAH, initiation of treatment with intravenous or subcutaneous prostanoids, lung transplantation, atrial septostomy, or all-cause death. Worsening of PAH was defined as the occurrence of all three of the following: a decrease in 6MWD of ≥ 15% from baseline, confirmed by a second 6MWD performed on a different day within 14 days; worsening symptoms of PAH; and the need for additional treatment for PAH. Worsening symptoms of PAH was defined as a change from baseline to a higher WHO functional class (or no change in patients who were in WHO functional class IV at baseline) plus the appearance or worsening signs of right heart failure not responsive to oral diuretic therapy. An independent clinical event committee adjudicated all deaths and reported events as to their relationship to PAH.

### Statistical analysis

Continuous variables were expressed as mean ± SD. Normality was examined using Kolmogorov–Smirnov and Shapiro–Wilk tests. Differences in continuous variables between groups were analyzed using *t* test or Wilcoxon's rank sum tests, as appropriate. Categorical variables were compared using Fisher's exact test. Event-free survival was estimated using the Kaplan-Meier method and differences between groups compared with the log-rank test. Univariate analysis was performed to determine if pericardial fluid amount was the independent factor of clinical events. Statistical significance was defined as a two-sided *P* value < 0.05. All analyses were performed using SPSS 16.0 (SPSS Institute Inc., Chicago, Ill., USA)

## CONCLUSIONS

The present study has demonstrated PE serves as a hallmark of severe progressive PAH. PADN results in significant absorption of PE with subsequent improvement of clinical outcomes. Multicenter randomized trials are warranted to determine the utility of PADN in high-risk patients with PAH.

## References

[1] Galie N, Hoeper MM, Humbert M, Torbicki A, Vachiery JL, Barbera JA, Beghetti M, Corris P, Gaine S, Gibbs JS, Gomez-Sanchez MA, Jondeau G, Klepetko W (2009). Guidelines for the diagnosis and treatment of pulmonary hypertension: The Task Force for the Diagnosis and Treatment of Pulmonary Hypertension of the European Society of Cardiology (ESC) and the European Respiratory Society (ERS), endorsed by the International Society of Heart and Lung Transplantation (ISHLT). Eur Heart J.

[2] Hinderliter AL, Willis PW, Long W, Clarke WR, Ralph D, Caldwell EJ, Williams W, Ettinger NA, Hill NS, Summer WR, de Biosblanc B, Koch G, Li S (1999). Frequency and prognostic significance of pericardial effusion in primary pulmonary hypertension. PPH Study Group. Primary pulmonary hypertension. Am J Cardiol.

[3] Batal O, Khatib OF, Dweik RA, Hammel JP, McCarthy K, Minai OA (2012). Comparison of baseline predictors of prognosis in pulmonary arterial hypertension in patients surviving ≤ 2 years and those surviving ≥ 5 years after baseline right-sided cardiac catheterization. Am J Cardiol.

[4] Honeycutt GR, Safdar Z (2013). Pulmonary hypertension complicated by pericardial effusion: a single center experience. Ther Adv Respir Dis.

[5] Zhang R, Dai LZ, Xie WP, Yu ZX, Wu BX, Pan L, Yuan P, Jiang X, He J, Humbert M, Jing ZC (2011). Survival of Chinese patients with pulmonary arterial hypertension in the modern treatment era. Chest.

[6] Batal O, Dardari Z, Costabile C, Gorcsan J, Arena VC, Mathier MA (2015). Prognostic Value of Pericardial Effusion on Serial Echocardiograms in Pulmonary Arterial Hypertension. Echocardiography.

[7] Benza RL, Miller DP, Gomberg-Maitland M, Frantz RP, Foreman AJ, Coffey CS, Frost A, Barst RJ, Badesch DB, Elliott CG, Liou TG, McGoon MD (2010). Predicting survival in pulmonary arterial hypertension: insights from the Registry to Evaluate Early and Long-Term Pulmonary Arterial Hypertension Disease Management (REVEAL). Circulation.

[8] Chen SL, Zhang FF, Xu J, Zhou L, Nguyen T, Stone GW (2013). Pulmonary artery denervation to treat pulmonary arterial hypertension: the single-center, prospective, first-in-man PADN-1 study (first-in-man pulmonary artery denervation for treatment of pulmonary artery hypertension). J Am Coll Cardiol.

[9] Chen SL, Zhang YJ, Zhou L, Xie DJ, Zhang FF, Jia HB, Wong SS, Kwan TW (2013). Percutaneous pulmonary artery denervation completely abolishes experimental pulmonary arterial hypertension *in vivo*. EuroIntervention.

[10] Breda AP, Pereira de Albuquerque AL, Jardim C, Morinaga LK, Suesada MM, Fernandes CJ, Dias B, Lourenço RB, Salge JM, Souza R (2014). Skeletal muscle abnormalities in pulmonary arterial hypertension. PLoS One.

[11] Nagaya N, Nishikimi T, Uematsu M, Satoh T, Kyotani S, Sakamaki F, Kakishita M, Fukushima K, Okano Y, Nakanishi N, Miyatake K, Kangawa K (2000). Plasma brain natriuretic peptide as a prognostic indicator in patients with pulmonary hypertension. Circulation.

[12] Ciarka A, Vachiery JL, Houssiere A, Gujic M, Stoupel E, Velez-Roa S, Naeije R, van de Borne P (2007). Atrial septostomy decreases sympathetic overactivity in pulmonary arterial hypertension. Chest.

[13] Velez-Roa S, Ciarka A, Najem B, Vachiery JL, Naeije R, van de Borne P (2004). Increased sympathetic nerve activity in pulmonary artery hypertension. Circulation.

[14] Folino AF, Bobbo F, Scgiraldi C (2003). Ventricular arrhythmias and autonomic profile in patients with primary pulmonary hypertension. Lung.

[15] Townsley MI (2012). Structure and composition of pulmonary arteries, capillaries, and veins. Comp Physiol.

[16] Richard JB (1979). Nerve supply to the lung. Am Rev Respir Dis.

[17] Swiston JR, Johnson SR, Granton JT (2010). Factors that prognosticate mortality in idiopathic pulmonary arterial hypertension: a systematic review of the literature. Respir Med.

[18] McLaughlin VV, Presberg KW, Doyle RL, Abman SH, McCrory DC, Fortin T, Ahearn G (2004). American College of Chest Physicians. Prognosis of pulmonary arterial hypertension: ACCP evidence-based clinical practice guidelines. Chest.

[19] Le Tourneau T, Richardson M, Juthier F, Modine T, Fayad G, Polge AS, Ennezat PV, Bauters C, Vincentelli A, Deklunder G (2010). Echocardiography predictors and prognostic value of pulmonary artery systolic pressure in chronic organic mitral regurgitation. Heart.

[20] Kainuma S, Taniguchi K, Toda K, Funatsu T, Kondoh H, Nishino M, Daimon T, Sawa Y (2010). Pulmonary hypertension predicts adverse cardiac events after restrictive mitral annuloplasty for severe functional mitral regurgitation. J Thorac Cardiovasc Surg.

[21] Austin C, Alassas K, Burger C, Safford R, Pagan R, Duello K, Kumar P, Zeiger T, Shapiro B (2015). Echocardiographic assessment of estimated right atrial pressure and size predicts mortality in pulmonary arterial hypertension. Chest.

[22] Galie N, Manes A, Negro L, Palazzini M, Bacchi-Reggiani ML, Branzi A (2009). A meta-analysis of randomized controlled trials in pulmonary arterial hypertension. Eur Heart J.

[23] Tonelli AR, Mubarak KK, Li N, Carrie R, Alnuaimat H (2011). Effect of balloon inflation volume on pulmonary artery occlusion pressure in patients with and without pulmonary hypertension. Chest.

[24] Borg GA (1982). Psychophysical bases of perceived exertion. Med Sci Sports Exerc.

[25] Lang RM, Bierig M, Deverus RB, Flachskampf FA, Foster E, Pellikka PA, Picard MH, Roman MJ, Seward J, Shanewise JS, Solomon SD, Spencer KT, Sutton MS (2005). Recommendations for chamber quantification: a report from the American Society of Echocardiography's Guidelines and Standards Committee and the Chamber Quantification Writting Group. J Am Soc Echocardiogr.

[26] Tei C, Nishmu RA, Seward JB, Tajik AJ (1997). Noninvasive Doppler-derived myocardial performance index: correlation with simultaneous measurement of cardiac catheterization measurements. J Am Soc Echocardiogr.

